# Effıcacy and safety of dapagliflozin on diabetic patients receiving high-doses of insulin

**DOI:** 10.12669/pjms.35.2.21

**Published:** 2019

**Authors:** Meltem Sertbas, Yasar Sertbas, Nalan Okuroglu, Ali Burkan Akyildiz, Seda Sancak, Ali Ozdemir

**Affiliations:** 1 *Meltem Sertbas, Department of Internal Medicine, Fatih Sultan Mehmet Education and Research Hospital, Istanbul, Turkey*; 2 *Yasar Sertbas Department of Internal Medicine, Fatih Sultan Mehmet Education and Research Hospital, Istanbul, Turkey*; 3 *Nalan Okurglu, Department of Internal Medicine, Fatih Sultan Mehmet Education and Research Hospital, Istanbul, Turkey*; 4 *Ali Burkan Akyildiz, Department of Internal Medicine, Fatih Sultan Mehmet Education and Research Hospital, Istanbul, Turkey*; 5 *Seda Sancak, Fatih Sultan Mehmet Education and Research Hospital, Istanbul, Turkey*; 6 *Ali Ozdemir, Department of Internal Medicine, Fatih Sultan Mehmet Education and Research Hospital, Istanbul, Turkey*

**Keywords:** Dapagliflozin, Efficacy, High dose insulin

## Abstract

**Objective:**

In this study we aimed to investigate the efficacy and safety of dapagliflozin addition to diabetic patients using high dose insulin.

**Methods:**

The current study was carried out in the outpatient diabetic clinics of Fatih Sultan Mehmet Education and Research Hospital. Thirty diabetic patients who were receiving high dose (>0,5U/kg) insulin and oral antidiabetic treatment (other than SGLT 2 inhibitors) were included in this study. Primary end point was the change in HbA1c, insulin doses and serum electrolyte from the addition of dapagliflozin 10 mg to the week 12.

**Results:**

At the end of three month BMI were obviously decreased from 33.31 ±4.51 to 32.14 ±4.66 (p: 0.001). There was also an evident decrease of insulin requirement from 76 ±23.15 U/kg to 57.60 ±17.61 U/day (p<0.001). As well as the decrease in insulin doses, there was also a significant decline in HbA1c (Δ 1.6 %) and fasting blood glucose levels (Δ68.6 mg/dl) (p<0.001). Among serum electrolyte levels slight but meaningful increase of blood urea nitrogen (BUN) and sodium (Na) levels were seen (p: 0.044 and p: 0.026). There were no significant changes in serum cholesterol levels with electrolytes such as potassium, calcium, phosphorus magnesium and vitamin D (p> 0.05).

**Conclusion:**

In diabetic patients with inadequately controlled glucose regulation despite high-dose insulin therapy, dapagliflozin may be an alternative combination choice to decrease the need of insulin dose and obtain an optimal HbA1c, fasting plasma glucose levels and weight without major side effects.

## INTRODUCTION

International Diabetes Federation (IDF) diabetes Atlas had estimated that in 2017 there were 451 million people with diabetes worldwide and expected to increase to 693 million by 2045.^1^ Diabetes mellitus is a chronic metabolic disease that requires lifelong follow-up and treatment, characterized by absolute or partial loss of insulin secretion or decrease of the peripheral effect of insulin. It has been associated with the development of both macrovascular and microvascular complications with the increase of morbidity and mortality.^2^ Less than 50% of the patients achieve glycemic targets with the oral antihyperglycemic agents and most of the patients require insulin therapy.^3^

Although insulin therapy is effective and the dose can be up titrated to address progressive deteoriations in glycemic control over time, higher doses of insulin might be owing to the risk of hypoglycemia, undesirable weight gain and fluid retention.^3^ Therefore, it is evident that there is an oral antidiabetic requirement besides insulin therapy to decrease the need of insulin doses.

Dapagliflozin is a potent, highly selective inhibitor of renal sodium glucose co-transporter 2 (SGLT2).^4^ Dapagliflozin increases urinary excretion of glucose by inhibiting renal glucose reabsorption thus decreasing plasma glucose. Since it acts independently from the insulin secretion or action, it provides additional glycemic control when used with insülin.^5^ SGLT2 inhibition may also affect sodium reabsorption and excretion, which may have effects on the renin-angiotensin system.^6^

The purpose of the present study was to investigate the efficacy and safety of dapagliflozin addition to diabetic patients using high dose insulin. Although a few studies have been performed in this area, when we search for clinical trials in PubMed (from inception through June 2016), no one have been investigated the effects of dapagliflozin on serum electrolytes and the insulin dose reduction on high-dose insulin using patients in the Turkish population.

## METHODS

The current study was carried out in the outpatient diabetic clinics of Fatih Sultan Mehmet Education and Research Hospital. Thirty diabetic patients who were receiving high dose (>0,5U/kg) insulin and oral antidiabetic treatment (other than SGLT 2 inhibitors) were recruited to this study. After the addition of dapagliflozin 10 mg to current treatments, three months later we investigated the HbA1c, insulin doses and serum electrolyte changes.

The present study was carried out in accordance with the ethical principles that have their origin in the Declaration of Helsinki, and approved by the Ethics Committee of the Fatih Sultan Mehmet Education and Research Hospital. Written informed consent was obtained from all participants (FSMEAH- 17073117-050.99).

### Patients

The patients aged between 18-75 years old with Type-2 diabetes receiving insulin >0,5U/kg for the past four weeks before enrollment and with HbA1c 7,5-12 %. Additional treatment with metformin and dipeptidyl peptidase-4 (DPP-4) inhibitor was allowed and patients with an estimated glomerular filtration rate (eGFR) of 60 ml/min/1,73 m^2^ and higher were enrolled. Patients having a history of Type-1 diabetes mellitus, cardiovascular events (acute coronary syndrome, unstable angina or myocardial infarction with hospitalization and acute stroke), breast feeding and pregnancy were excluded from the study.

During the 12-weeks followup period, a fixed dose insulin regimen was tried to apply in order to evaluate the efficacy of dapagliflozin to see the changes of HbA1c, insulin dose and serum electrolyte levels accurately unless there was obvious clinical indication for the down titration as follows: hypoglycemic symptom with self-monitoring of blood glucose (SMBG) <70 mg/dLwithout drastic changes in daily life/activities or the investigators judge patients at a high risk of hypoglycemia.

### Statistical Analysis

SPSS 22 for Windows statistical program was used for statistical analyses. The results of all parameters belonging to patients were given as mean ± standard deviation. The dependent T test was used for parametric data to analyze values before and after dapagliflozin treatment. Wilcoxon test was used for the data which were not compatible with parametric distribution. The statistical significance level of the data obtained was interpreted using “p” values. A p value of < 0.05 was considered statistically significant. The Kolmogorov Smirnov test was used to determine parametric and non-parametric distributions of the data. The data with a p value of > 0.05 were considered to have parametric distribution and values below this were considered non-parametric values.

## RESULTS

Thirty diabetic patients (19 women and 11 men) who had high dose insulin usage (> 0,5 U/kg, total insulin dose: 76±23.15) were included in this study. The average age was 57,73 ± 6,13 and the duration of diabetes in patients was 11,46 ± 6,7. We investigated the changes in the insulin requirements, blood pressure levels, body mass index (BMI) and biochemical parameters between the time of admission and at three months after the application of dapagliflozin treatment.

After three months of dapagliflozin usage, BMI of the patients was significantly decreased from 33.31 ±4. 51 to 32.14 ±4. 66 (p: 0.001). There was also an obvious decrease of insulin as the total daily insulin requirement decreased from 76 ±23.15 U/kg to 57.60 ±17.61 U/day (p<0.001). Apart from the decrease in insulin doses, there was also a significant decline in HbA1c (Δ 1.6 %) and fasting blood glucose levels (Δ68.6 mg/dl) (p<0.001) (Table-I).

**Table-I:** Effect of dapagliflozin on glucose regulation at 12 week.

	Basal	3 monthlater	P value
BMI (kg/m^2^)	33.31±4.51	32.14±4.66	< 0.001
FPG (mg/dl)	234.7±67.89	166.07±43.93	< 0.001
HbA1c (%)	9.67±1.44	8.07±1.15	< 0.001
Total insulin dose (U/day)	76±23.15	57.60±17.61	< 0.001

Although the serum hematocrite level was increased, it was not statistically meaningful. On the other hand the serum BUN levels were significantly increased from 14.90±3.17 to 16.66±0.54 (p: 0.044). Among serum electrolytes, serum potassium levels were increased slightly (p> 0.05). Serum Na is the other electrolyte that the levels increased meaningfully (p: 0.026).

Serum calcium, phosphor and magnesium levels were also increased, but not as high as to be statistically significant (p> 0.05). There were non-significant decrease of serum uric acid levels (p>0.05) (Table-II). Among lipid parameters there were non-significant changes of LDL and HDL were seen. Although there was an obvious decrease in triglyceride level, it was not statistically significant (p: 0.136) (Table-III).

**Table-II:** Effect of dapagliflozin on serum hemogram and electrolytes.

	Basal	3 month later	P value
Hemoglobin (g/dL)	13.64±1.39	13.58±1.26	0.70
Hematocrite (%)	41.23±4.28	41.59±4.53	0.47
Sodyum (mEq/L)	138.23±2.64	139.40±1.95	0.03
Potasyum (mEq/L)	4.70±0.53	4.71±0.37	0.88
Klorür (mEq/L)	102.70±2.72	102.13±2.27	0.28
BUN (mEq/L)	14.90±3.17	16.66±0.54	0.04
Creatin (mEq/L)	0.81±0.11	0.84±0.12	0.15
GFR (mL/min/1.73 m^2^)	84.00±12.68	81.87±11.27	0.17
Kalsiyum (mg/dL)	9.53±0.57	9.62±0.40	0.44
Phosphate (mg/dL)	3.89±0.76	4.02±0.63	0.48
Vitamin D (ng/ml)	18.61±8.31	19.01±10.05	0.36
Magnesium (mg/dL)	1.74±0.27	1.81±0.25	0.20
Urik asit (mg/dL)	5.15 ±1.41	4.86±1.46	0.23

**Table-III:** Effect of dapagliflozin on lipid parameters.

	Basal	3 month later	P value
LDL (mg/dL)	121.66±34.29	123.56±33.96	0.81
HDL (mg/dL)	38.13±7.82	37.38±7.64	0.45
Trigliserid (mg/dL)	199.23±100.01	175.89±66.83	0.14

## DISCUSSION

Diabetes Mellitus (DM) is a progressive disease which often requires insulin therapy during the course of the disease. Hypoglycemia, weight gain and fluid retention are the significant factors that limit optimal titration and effectiveness of insulin.^7^ Therefore, there is still a need of medical requirement for Type-2 diabetes mellitus even with insulin therapy. Agents that selectively block sodium glucose cotransporter 2 (SGLT2), located in the proximal tubule of the kidney inhibit glucose absorption, induce its elimination and lowers blood glucose independently of insulin.^8^,^9^

In this study we evaluated the safety and efficiency of the dapagliflozin on the patients using high dose insulin. Several studies have shown that the elimination of glucose through urine by SGLT2 inhibitors leads to a loss of calories around 200-300 kcal/day, resulting in a negative energy balance.^10^ We have seen a significant weight reduction in the patients recruited to this study (88.51 ± 13.41 kg to 85.35 ± 13.69 kg with BMI Δ:1.17kg/m^2^ p<0.005). Due to negative energy balance, urinary glucose excretion may be the cause for the loss of calories and studies consistently shown weight loss usually 2-4 kg.^11^,^12^

In clinical trials dapagliflozin improved glycemic control and reduced HbA1c and fasting plasma glucose when administered with insulin.^11^,^12^ Although after addition of dapagliflozin Wilding et al. decreased the insulin dose half of the basal level, they reached a decrease of 0.61% HbA1c, FPG Δ: 4.3mg/dl.^11^ In another study with the addition of dapagliflozin to insulin in Japanese patients, there was a significant change of HbA1c (8.26% to 7.54) and fasting plasma glucose (FPG Δ: - 21.7mg/dl) but the decrease of the mean daily insulin dose was not significant (-0.74 IU/day).^7^ In our study there was an obvious decrease of all the three parameters as FPG from 234.7 ±67. 89 to 166.07 ±43.93, HbA1c from 9.67 ±1.44 to 8.07 ±1. 15 and insulin doses from 76 ±23.15 to 57.60 ±17.61 (p< 0.001).

As seen in several studies that the hematocrit level slightly increase, in our study there were also slightly, but non-significant increase of hematocrit level were found.^10^-^12^ James et al. showed that dapagliflozin exhibited a glucose induced osmotic diuresis with a small increase in BUN, and small dose dependent increase in hematocrit levels. There were no clinically meaningful change of serum creatinine and estimated glomerular filtration rate.^13^

SGLT2 inhibitor administration, mainly canagliflozin, is associated with small increases in serum potassium concentration, especially in patients with reduced renal function.^14^ No change in serum potassium levels was also found with empagliflozin in the EMPA-REG trial and with dapagliflozin which was not associated with serum potassium changes in patients with moderate renal impairment (eGFR 30–59 ml/min/1.73m^2^).^15^,^16^ In our study we showed a slight but nonsignificant elevation of potassium levels in agreement with the literatures. Increased glucagon levels and osmotic diuresis SGLT2 inhibitors may cause a slight decrease of potassium levels, which in turn increases serum potassium levels with redistribution due to decreased insulin levels.^17^

Eighteen randomized controlled meta-analysis with SGLT2 inhibitors including dapagliflozin have showed that these drugs dose dependency can increase magnesium levels by approximately 0.08-0.2 mEq/L in individuals without kidney disease.^18^ In our study we have also seen a slight increase in accordance with literature. It has been claimed that the increase in serum magnesium along with an increase in serum potassium concentrations may decrease the risk of cardiac arrhythmias, a possible beneficial effect explaining, at least in part, the cardiovascular event reduction in the EMPA-REG OUTCOME and Canagliflozin Cardiovascular Assessment Study (CANVAS) trials.^17^

Uric acid is the end product of purine metabolism. Hyperuricemia in addition causing gout, is also independently associated with increased risk of cardiovascular outcomes.^19^ Reduction of serum uric acid levels has been seen with SGLT2 inhibitors due to increased urinary excretion.^20^ In our study, we observed a statistically nonsignificant slight decrease of serum uric acid levels.

In a study with a moderate renal impairment, 9.4% of patients treated with dapagliflozin (10 mg) experienced bone fractures while no fractures were observed in placebo-treated patients.^21^ Furthermore, a ~30% increase in bone fractures was observed in canagliflozin-treated patients in eight pooled clinical trials with a longer mean duration (68 weeks).^22^ Possible mechanism of that were explained as the increase of serum phosphate is followed by an increase in parathormone (PTH) levels and additionally hyperphosphatemia associated with the increase of fibroblast growth factor 23 (FGF-23) which may decrease Vitamin D concentrations leading to decreased bone mineral density.^23^ However, other studies have not confirmed the adverse effects on calcium homeostasis or on hormones regulating calcium levels, and they showed that the decrease in BMD was obviously correlated with weight loss.^24^,^25^ In our study there were no significant change of serum phosphate, calcium and Vitamin D levels after the use of dapagliflozin for 12 weeks.

There are conflicting results regarding the effects of this class of medicines on lipid parameters. One of the studies showed a dose-dependent increase with canagliflozine, but no significant effect on LDL cholesterol was also observed in another study conducted with dapagliflozin.^20^ We have also seen a mild increase of LDL cholesterol with nonsignificant decrease of HDL and triglyceride levels.

The most common adverse events occurring with dapagliflozin are urinary tract infection (UTI) and genital tract infections (GTI) related with glycosuria.^4^ In the metanalysis of Yingying et al. GTI occurred more often compared to placebo, but UTI has a small increase in SGLT 2 groups with no statistical significance.^5^ During our study there was no case of UTI and only one patient with GTI was seen and responded standard therapy while continuing to dapagliflozin. We also didn’t see any major hypoglycemic events during the 3 month period of follow up.

In conclusion, in diabetic patients with inadequately controlled glucose regulation despite high-dose insulin therapy, dapagliflozin may be an alternative combination choice to decrease the need of insulin dose and obtain an optimal HbA1c, fasting plasma glucose levels and weight without major side effects.

### Authors Contribution

**MS, YS, AO:** Conceived, designed and did statistical analysis.

**YS, NO, ABA, SS:** Did data collection and manuscript writing.

**YS, AO:** Did review and final approval of manuscript.
